# Pilonidal Sinus of the Penis

**DOI:** 10.1100/tsw.2004.74

**Published:** 2004-06-07

**Authors:** Hugh F. O'Kane, B. Duggan, C. Mulholland, J. Crosbie

**Affiliations:** Department of Urology, Altnalgelvin Hospital Northern Ireland, UK

## Abstract

A pilonidal sinus is a subcutaneous sinus containing hair. It is most commonly found in the natal cleft of hirsute men. Here we describe the unusual finding of a pilonidal sinus arising on the male foreskin.

## CASE REPORT

A 61 year old man presented with a penile ulcer which had been present for several years. An indurated skin ulcer was found on the ventral aspect of the prepuce, with an associated tight phimosis. A dorsal slit revealed extensive hair nests and sinuses requiring excision and circumcision.(Figure 1) Histology revealed a tract partially lined with squamous epithelium containing keratin and hair associated with granulation tissue and giant cells - appearances consistent with pilonidal sinus. (Figure 2)

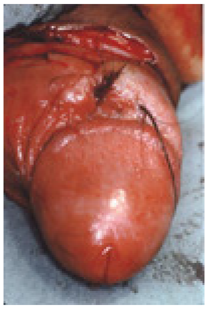

**FIGURE 1**. Nests of hair found beneath foreskin.

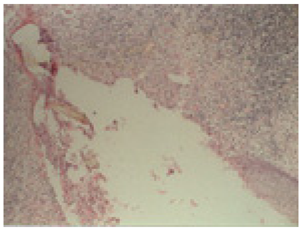

**FIGURE 2**. Histology showing sinus containing hair fragments.

## DISCUSSION

The term "pilonidal sinus" (derived from the latin; pilus - a hair and nidus - a nest), first introduced in 1880,(1) may be used to describe any subcutaneous sinus which contains hair. Although most commonly found in the natal cleft of hirsute males, less common sites include the umbilicus, axilla and the web spaces of the fingers. Pathogenesis of the disease was accepted as having a congenital origin for many years originating in an underlying cutaneous pit or subcutaneous inclusion sacrococcygeal cyst. Another theory suggests that the sinus starts by penetration of the skin by hairs.(2) The occasional finding of hairs not originating from the area of the particular lesion would support this. Pilonidal sinus on the penis is extremely rare, with only six cases reported in the literature. It appears exclusively to occur in uncircumcised men and invariably is associated with a phimosis. The lesion usually presents following recurrent episodes of infection, An association with Actinomycosis has been described.(3) Treatment should involve excision and circumcision. Associated carcinoma should be excluded on histology.

## References

1. Hodges RM: In: Burnand K, Young A, editors. New Aird's Companion in Surgical Studies. 2nd ed. United Kingdom: Churchill Livingstone; 1998, p. 819.
2. Patey DH: A reappraisal of the acquired theory of sacrococcygeal pilonidal sinus and an assessment of its influence on surgical practice. Br J Surg, 1969; 56(6):463-6.
3. Rashid AMH, Menai Williams R, Parry D, Malone PR: Actinomycosis associated with pilonidal sinus of the penis. J Urol, 1992; 148:405-6.
